# Rotational switches in the two-dimensional fullerene quasicrystal

**DOI:** 10.1107/S2053273318015681

**Published:** 2019-01-01

**Authors:** M. Paßens, S. Karthäuser

**Affiliations:** a Peter Grünberg Institut (PGI-7) and JARA-FIT, Forschungszentrum Jülich GmbH, Jülich 52425, Germany

**Keywords:** rotational switches, fullerenes, interfacial interactions, geometric frustration, dodecagonal quasicrystals, square–triangle tiling, scanning tunneling microscopy

## Abstract

One of the essential components of molecular electronic circuits are switching elements that are stable in two different states and can ideally be switched on and off many times. Here, distinct buckminsterfullerenes within a self-assembled monolayer, forming a two-dimensional dodecagonal quasicrystal on a Pt-terminated Pt_3_Ti(111) surface, are identified to form well separated molecular rotational switching elements. Employing scanning tunneling microscopy, the molecular-orbital appearance of the fullerenes in the quasicrystalline monolayer is resolved. Thus, fullerenes adsorbed on the 3^6^ vertex configuration are identified to exhibit a distinctly increased mobility. In addition, this finding is verified by differential conductance measurements. The rotation of these mobile fullerenes can be triggered frequently by applied voltage pulses, while keeping the neighboring molecules immobile. An extensive analysis reveals that crystallographic and energetic constraints at the molecule/metal interface induce an inequality of the local potentials for the 3^6^ and 3^2^.4.3.4 vertex sites and this accounts for the switching ability of fullerenes on the 3^6^ vertex sites. Consequently, a local area of the 8/3 approximant in the two-dimensional fullerene quasicrystal consists of single rotational switching fullerenes embedded in a matrix of inert molecules. Furthermore, it is deduced that optimization of the intermolecular interactions between neighboring fullerenes hinders the realization of translational periodicity in the fullerene monolayer on the Pt-terminated Pt_3_Ti(111) surface.

## Abstract 2

Local potential differences between the 3^6^ and 3^2^.4.3.4 vertex configurations are identified within a two-dimensional dodecagonal fullerene monolayer. In a local area of the 8/3 approximant, rotational switching fullerenes on 3^6^ vertex sites are revealed by scanning tunneling microscopy.

## 1. Introduction   

One of the key challenges in molecular electronics design is the stability of molecular states that are generally triggered from an ‘on state’ to an ‘off state’ or vice versa by application of an optical, magnetic or electric pulse (Zhang *et al.*, 2015 ▸; Jeong *et al.*, 2017 ▸). One way to achieve this goal is to use intrinsically bistable molecules which can be employed as switching units without major changes in their dimensions or electronic properties. Furthermore, they need to be adsorbed on a flat surface and wired to two metallic electrodes. Some prominent molecule families that are known to fulfill these conditions to a large extent are di­aryl­ethenes (Irie *et al.*, 2014 ▸; Reecht *et al.*, 2017 ▸), azo­benzenes (Alemani *et al.*, 2006 ▸), and catenanes and rotaxanes (Dichtel *et al.*, 2007 ▸; Fahrenbach *et al.*, 2013 ▸). Their ability to switch in a controlled manner between two states by conformation change, ring opening/closing or redox reaction has been thoroughly studied in self-assembled monolayers or as single molecules using scanning tunneling microscopes, in break junctions, and in device geometries (van der Molen & Liljeroth, 2010 ▸; Feringa & Browne, 2011 ▸; Song *et al.*, 2011 ▸; Jia *et al.*, 2016 ▸). However, the problems with intrinsically bistable molecules are their often complicated chemical synthesis, the change in molecular properties caused by adsorption on a substrate, and an energetic disparity between the on and off states. A limited device performance, such as a degradation of the reliability or retention, often results.

Alternatively, a switching unit can be formed by a simple molecule or even an atom in interaction with a surface, if a double-well potential can be successfully created during adsorption (Eigler *et al.*, 1991 ▸; Loth *et al.*, 2012 ▸). The same effect is achieved by providing energetically comparable adsorption sites on the surface that can be probed by one molecule. If these adsorption sites are quite dense, a molecule can jump from one site to another after being triggered by an electrical pulse. In addition, the molecule may also change its adsorption configuration or rotate while staying on the same adsorption site (Godlewski *et al.*, 2016 ▸; Perera *et al.*, 2013 ▸; Wasio *et al.*, 2017 ▸). It has been possible to explore these new aspects of molecular electronics following the enormous advances in scanning probe microscopy (SPM) in recent decades, which have allowed the characterization, addressing or manipulation of atoms and molecules with atomic scale precision in a controlled manner (Stroscio & Eigler, 1991 ▸; Ebeling *et al.*, 2017 ▸; Cirera *et al.*, 2017 ▸; Gross *et al.*, 2018 ▸).

Buckminsterfullerenes (C_60_ fullerenes) are considered ideal probes to test the properties of solid surfaces. They are composed of carbon, and possess an icosahedral cage, high symmetry and high rigidity. Moreover, their orbital structure can be resolved by scanning tunneling microscopy (STM), which makes it possible to identify the adsorption configuration on a substrate. Consequently, C_60_ fullerenes can be used as sensors to reveal interfacial interactions between molecules and atomically flat surfaces (Frederiksen *et al.*, 2014 ▸; Paßens *et al.*, 2015 ▸; Ledieu *et al.*, 2017 ▸). Thus, fullerenes should also be ideally suited to probing the broad ensemble of adsorption sites on the surfaces of quasicrystalline metallic alloys (Thiel, 2008 ▸). Indeed, using fullerenes or other molecules with π orbitals able to interact with metallic *d* states, quasicrystalline molecular monolayers have been assembled using ternary metallic alloys with quasicrystalline order as the template (Smerdon *et al.*, 2014 ▸; Kalashnyk *et al.*, 2018 ▸). Other two-dimensional molecular quasicrystals have been created based on intermolecular hydrogen bonding (Wasio *et al.*, 2014 ▸) or metal–organic network formation (Urgel *et al.*, 2016 ▸).

Recently, we introduced the formation of a two-dimensional fullerene quasicrystal (QC) by self-assembly of fullerenes on a Pt alloy surface (Paßens *et al.*, 2017 ▸). The employed Pt_3_Ti(111) alloy single crystal was terminated by two layers of Pt [2Pt–Pt_3_Ti(111)], resulting in an electronically structured surface as indicated by STM and *ab initio* density functional theory (DFT) calculations (Paßens *et al.*, 2016 ▸). The complex substrate-related adsorption-energy landscape with respect to C_60_ was identified as the driving force for the formation of the dodecagonal quasicrystalline fullerene monolayer. Further, a random square–triangle tiling with phason strain enables the accommodation of the two-dimensional quasicrystalline structure on the periodic 2Pt–Pt_3_Ti(111) substrate (Paßens *et al.*, 2017 ▸). Since this quasicrystalline system is rather simple with respect to dimensionality (two-dimensional), molecular compound (one compound, highly symmetric, only composed of carbon), substrate surface (hexagonal structure) and tiling (triangles and squares), investigations of the interactions of fullerenes with the 2Pt–Pt_3_Ti surface provide an opportunity to collect some hints to unanswered questions concerning QC formation and stability (Steurer, 2018 ▸; Savitz *et al.*, 2018 ▸).

Here, we will examine a highly efficient molecular switch, which can be created by the interaction of easily accessible molecules with a surface providing a variety of adsorption possibilities. In detail, we will demonstrate that buckminster­fullerenes adsorbed on the 2Pt–Pt_3_Ti(111) alloy surface fulfill the prerequisites for a molecular switching system by using ultra-high-vacuum (UHV) SPM methods. Moreover, we will identify the switching ability of individual fullerenes as an intrinsic property of the two-dimensional fullerene QC created by adsorption on the periodic alloy surface.

## 2. Experimental details   

A Pt_3_Ti(111) single crystal was purchased from MaTecK (Germany) and prepared by several ion sputtering (3 × 10^−5^ mbar Ne^+^ at 1 kV, 10 min) and annealing (1200 K, 25 min) cycles under UHV conditions. Under these conditions an overlayer consisting of two Pt layers is formed on the Pt_3_Ti(111) surface (Paßens *et al.*, 2016 ▸). The cleanliness of the surface was checked by low-energy electron diffraction (LEED) and low-temperature ultra-high-vacuum (LT-UHV) STM.

Organic molecular-beam deposition (deposition rate 0.04 ml min^−1^, Knudsen cell at 593 K) was used for the self-assembly of C_60_ molecules (Sigma–Aldrich, purity 99.9%) on the 2Pt–Pt_3_Ti(111) substrate held at 323 K in UHV (Paßens *et al.*, 2017 ▸).

LT-UHV-STM (1 × 10^−10^ mbar) and scanning tunneling spectroscopy (STS) were performed with a commercial Createc instrument (Germany). STM measurements were usually performed at 77 K in constant-current mode using electrochemically etched tungsten tips. Differential conductivity spectra were recorded after switching off the feedback loop using lock-in detection of the a.c. tunneling current (modulation frequency 471 Hz, modulation amplitude 30 mV). During the switching measurements the feedback loop was turned off to maintain a constant tip-to-sample distance, while a certain bias voltage was applied.

## 3. Details of the adsorption geometry of C_60_ on 2Pt–Pt_3_Ti(111)   

**Figure 1:**
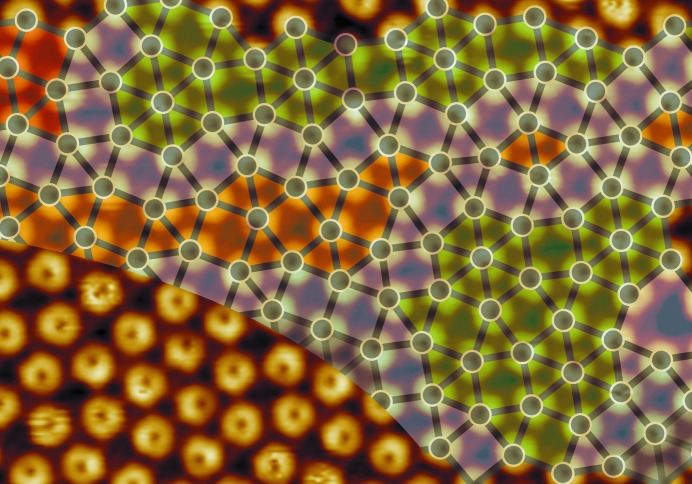
A UHV-STM image (13 nm × 9 nm, *U*_bias_ = −2.03 V, *I*_set_ = 0.47 nA) of the quasicrystalline fullerene monolayer superimposed by a color-coded tiling representation: 8/3 approximant in green, 3^2^.4.3.4 approximant in orange and red, and 3^6^ approximant in brown. The transition regions between the approximants are given in light gray.

As recently reported, the self-assembly of fullerenes on the 2Pt–Pt_3_Ti(111) alloy surface results in the formation of a two-dimensional dodecagonal QC (Paßens *et al.*, 2017 ▸). The real-space LT-UHV-STM image of the quasicrystalline monolayer is depicted in Fig. 1 ▸. It is composed of triangles and squares and is superimposed with the tiling representation. The color coding of this square–triangle tiling indicates local areas of the 8/3 approximant, the 3^2^.4.3.4 approximant and the 3^6^ approximant. The approximants are randomly distributed, and interpenetrating dodecagons and a small number of defects can also be detected. Consequently, the present dodecagonal QC corresponds to a random square–triangle tiling. Furthermore, the Γ^1b^ phason strain, which describes the local deviation from ideal quasicrystalline order, was deduced from spot shifts identified in the fast Fourier transform (FFT) spectrum of the quasicrystalline fullerene monolayer (Paßens *et al.*, 2017 ▸). Thus, fullerenes deposited on the 2Pt–Pt_3_Ti(111) single crystal form a two-dimensional dodecagonal QC with evident phason strain, while fullerenes normally form periodic structures on the (111) surfaces of metals including Pt.

**Figure 2:**
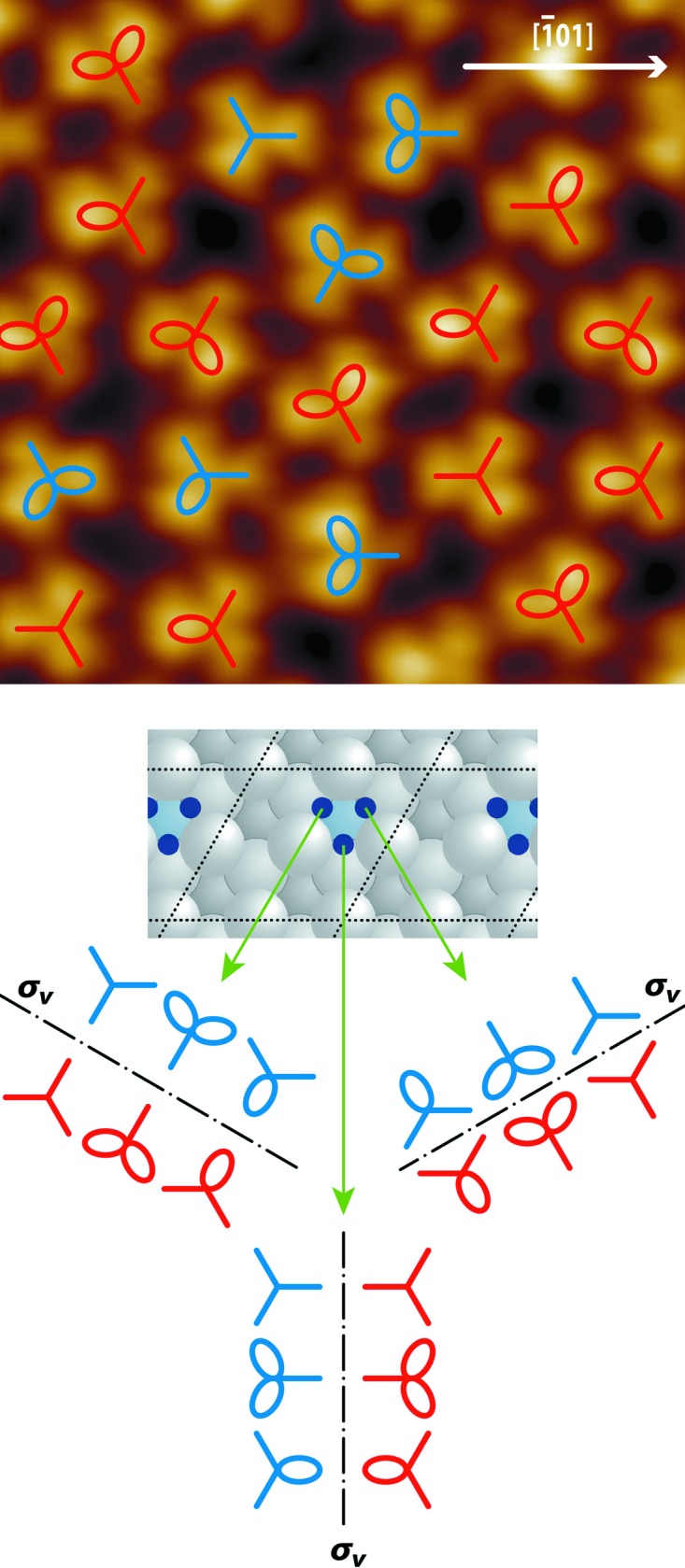
(Top) A UHV-STM image of fullerenes assembled on the 2Pt–Pt_3_Ti(111) surface (4.5 nm × 4.5 nm, *U*_bias_ = 2.22 V, *I*_set_ = 2.9 nA) overlaid by marks representing the tilting directions. (Middle) A schematic diagram representing the 2Pt–Pt_3_Ti(111) surface, with energetically preferred bridge adsorption sites indicated as dark-blue circles within the unit cell (black dotted lines). (Bottom) The marks schematically displaying the appearance of the three-lobe structure of fullerene as a result of the mirror plane and tilting for each of the three preferred adsorption sites in one unit cell.

In Fig. 1 ▸, the fullerenes are imaged at negative bias voltage, revealing the occupied states with the characteristic dip in the middle of the molecule. From large real-space images obtained at comparable set points and from FFT spectra, the mean intermolecular distance in this quasicrystalline monolayer *d*_C60—C60_ = 1.04 ± 0.03 nm was determined. It is slightly increased compared with the equilibrium intermolecular distance of 1.00 nm realized in the fullerene bulk crystal corresponding to the van der Waals radius (Krätschmer *et al.*, 1990 ▸). At positive bias voltage, when the electrons are tunneling from the STM tip into the surface, the appearance of the empty states of the molecules is mapped. The π electrons of fullerenes are not completely delocalized, and thus a small disparity in electron density remains between the C—C bonds with a slight single-bond character (0.145 nm) forming the pentagons and the bonds with a slight double-bond character (0.14 nm) located between two hexagons of the C_60_ cage (Lu *et al.*, 1992 ▸; Shi *et al.*, 2012 ▸; Gross *et al.*, 2012 ▸). Consequently, the empty states of the fullerenes are located at the positions of the pentagons and appear as bright spots in STM images at positive bias voltage (Wang *et al.*, 2001 ▸). They can be used to identify the orientation of the fullerenes on the surface. If a fullerene is adsorbed with a hexagon parallel to a metallic surface, the three pentagons surrounding the hexagon form a three-lobe structure. Despite the fact that fullerenes can adsorb in different orientations on metal surfaces due to their multiple rotational degrees of freedom, only the three-lobe structure is observed on the 2Pt–Pt_3_Ti(111) surface (Fig. 2 ▸), indicating a considerable interfacial interaction.

To analyze the adsorption-energy landscape, we have recently performed DFT calculations on these interfacial interactions between fullerenes and the 2Pt–Pt_3_Ti(111) surface (Paßens *et al.*, 2017 ▸). The degeneracy of the bridge sites on the outermost Pt layer was lifted due to the influence of the Ti atoms in the third atomic layer of the 2Pt–Pt_3_Ti(111) single crystal. Thus, three different types of bridge site exist on this surface. Furthermore, a threefold-degenerate bridge site was identified as an energetically preferred adsorption site for fullerenes. This bridge site is located between two surface Pt atoms next to one Ti atom in the third atomic layer, as marked by dark-blue circles in the middle part of Fig. 2 ▸.

Having examined the STM image of the three-lobe structure of the fullerenes (Fig. 2 ▸, upper part), the following points can be concluded based on the crystallographic symmetry of the preferred adsorption sites. First, the fullerenes exhibit rotational order: their three-lobe structure is aligned along the 〈
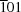
〉 directions. However, two different orientations rotated by 60° are possible, indicated by blue and red marks in the upper part of Fig. 2 ▸. A statistical analysis of larger images reveals that the fullerenes are equally distributed between these two directions. These two energetically equivalent orientations are a consequence of mirror planes perpendicular to the 〈
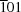
〉 directions located on the positions of the preferred bridge sites. Second, almost all fullerenes exhibit the three-lobe structure. However, not all three lobes display the same brightness. In some cases one, or in other cases two, lobes are brighter than the others. This is a result of the tilting of the respective fullerene towards one of the Pt atoms next to the bridge position. A slight tilting is energetically preferable to optimize the molecule–substrate inter­actions (Paßens *et al.*, 2017 ▸). However, it is also a mechanism to adapt the intermolecular distance at a low energy cost. The combination of the ability to tilt in the direction of a nearby Pt atom with the existence of the mirror plane on the site of the bridge position leads to the possible orbital appearances given in the lower part of Fig. 2 ▸. Interestingly, all orbital appearances of the tilted fullerenes are unique. They can be used to identify the adsorption on one distinct bridge site in the unit cell and the direction of the tilt of the fullerene to reduce the intermolecular distance.

**Figure 3:**
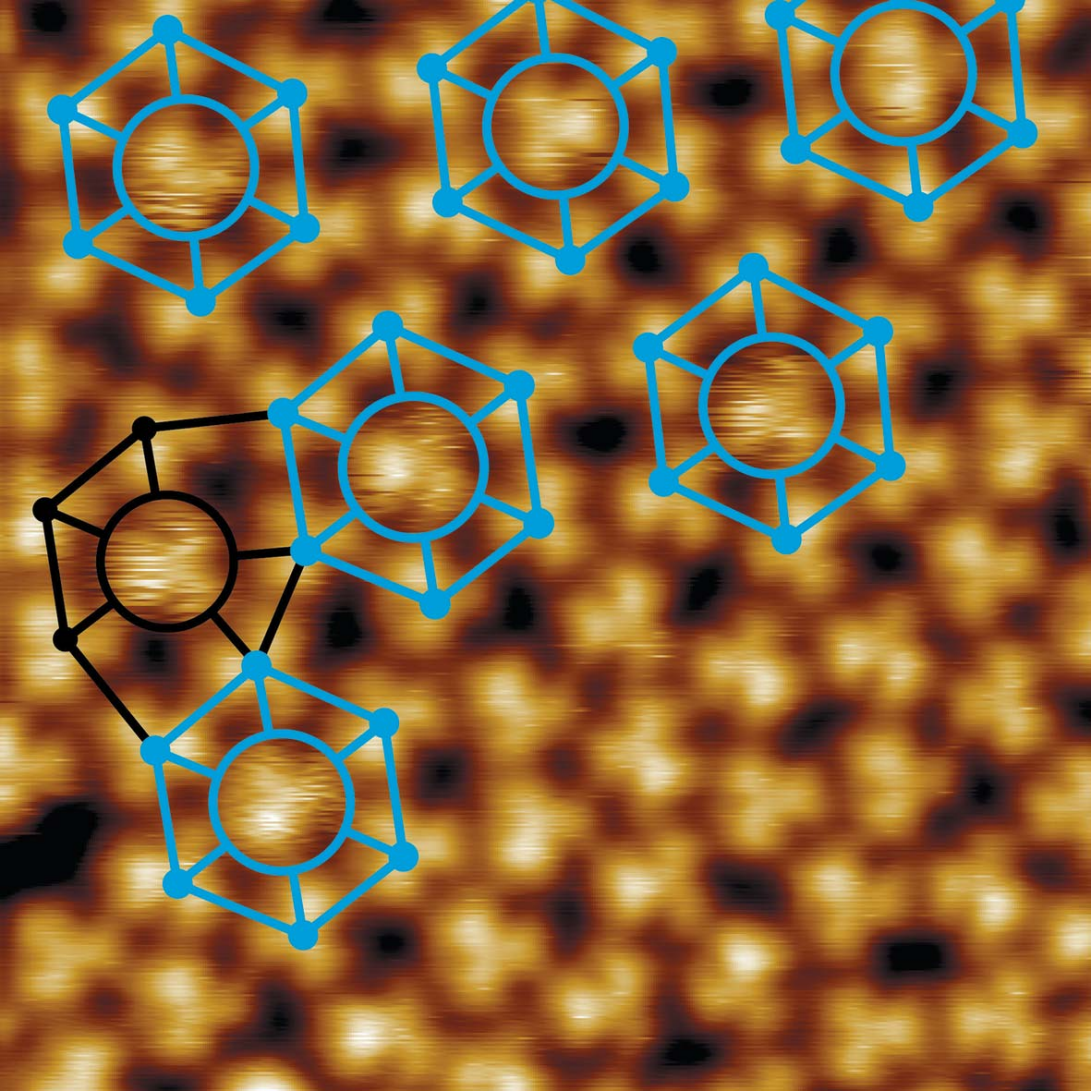
A high-resolution UHV-STM image of a local area of the quasicrystalline monolayer covered by the dodecagonal 8/3 approximant (8.4 nm × 8.4 nm, *U*_bias_ = 2.22 V, *I*_set_ = 0.59 nA, only plane corrected). The local vertex configuration of fullerenes exhibiting higher mobility is highlighted (3^6^ vertex configuration in blue, 3^2^.4.3.4 vertex configuration in black).

In Fig. 3 ▸, a plane-corrected STM image of the local area of the dodecagonal 8/3 approximant is shown. Here some ‘fuzzy’ fullerenes can be identified. A molecule appears fuzzy in an STM image if it moves while the image is being recorded. Obviously, there are fullerenes with a noticeably higher mobility. The local vertex configuration of these fuzzy fullerenes can be identified to be the hexagon (3^6^) in the center of the 8/3 approximant. In Fig. 3 ▸ a total of seven hexagons are present and six of them exhibit a central fullerene with an increased mobility. Only one fuzzy fullerene with the vertex configuration 3^2^.4.3.4 exists, shown in Fig. 3 ▸, *i.e.* one fullerene out of 61 with this vertex configuration. Thus, we conclude that the local potentials for each vertex site differ considerably in this two-dimensional fullerene QC.

Comparable observations have been made for quasi­periodic electronic states measured in a synthetic Penrose tiling (Collins *et al.*, 2017 ▸). In this case, carbon monoxide molecules were assembled by atomic manipulation using STM on a Cu(111) surface so that a Penrose tiling resulted. Indeed, the local vertex structures of the synthetic QC exhibited slightly different local potentials as measured by differential conductance spectroscopy.

## 4. Scanning tunneling spectroscopy   

**Figure 4:**
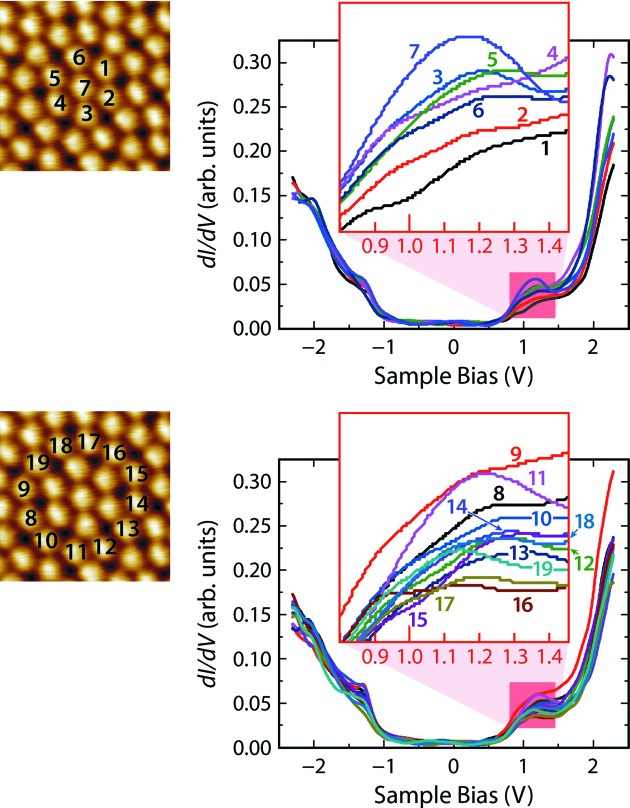
Differential conductance spectra (d*I*/d*V*) (*U*_bias_ = −2.42 V, *I*_set_ = 2.42 nA) over fullerenes, (top) in the inner hexagon and (bottom) in the outer ring of an 8/3 approximant. The insets show enlargements of the spectra in the voltage range around the LUMOs. (Left-hand side) Identification of the respective fullerenes in the STM image (6 nm × 6 nm, *U*_bias_ = −2.03 V, *I*_set_ = 0.28 nA).

To verify local potential differences between the fullerenes on the 3^6^ and 3^2^.4.3.4 vertex sites we performed STS on each fullerene of a dodecagon. Differential conductance spectra taken over the molecules represent their local density of states and can thus be used to determine the respective highest occupied molecular orbital–lowest unoccupied molecular orbital (HOMO–LUMO) gap and the interfacial interaction. The HOMO–LUMO gap of single fullerenes in the gas phase amounts to 4.9 V. However, it decreases strongly to 3.7 V in the bulk crystal, to 2.7 V on metal surfaces like Au(111) (Paßens *et al.*, 2015 ▸) and to only 2.5 V on 2Pt–Pt_3_Ti(111) (Fig. 4 ▸), indicating the strong interface interaction. Increased screening due to neighboring molecules and, more efficiently, due to interaction with metal surfaces leads to a reduction in the HOMO–LUMO gap (Torrente *et al.*, 2008 ▸). Moreover, strong interfacial interactions between metal *d* states and the threefold-degenerate LUMOs of fullerenes may induce a lifting of the degeneracy and further broadening of the LUMO levels (Paßens *et al.*, 2015 ▸).

In Fig. 4 ▸ the differential conductance spectra of all fullerenes forming an 8/3 approximant are shown. The LUMO can be identified at ∼1.2 V above the Fermi level, while the LUMO+1 is above 2 V. Below the Fermi energy, features corresponding to HOMO and HOMO−1 arise at around −1.30 V and around −2.05 V, respectively. The spectra, especially around the LUMO, are not perfectly aligned. We attribute this to small differences in the C_60_/2Pt–Pt_3_Ti(111) interfacial interactions. The enlargement of the differential conductance spectra (Fig. 4 ▸ top, inset) reveals that the LUMO states of almost all fullerenes of the hexagon are broadened and indications for a lifting of the degeneracy are present. The same is true for the LUMO states of the fullerenes located in the outer ring of the dodecagon (Fig. 4 ▸ bottom, inset). However, one differential conductance spectrum exhibits a considerably distinct LUMO, namely curve number 7, attributed to the central fullerene of the inner hexagon. It is the only one with a 3^6^ vertex configuration and obviously, at this position, the interfacial interaction is slightly less compared with fullerenes with the 3^2^.4.3.4 vertex configuration. This is in accordance with the fuzzy appearance of the fullerenes in the 3^6^ vertex configuration in the STM images and verifies the assumption that the local potentials for each vertex site differ considerably. Most importantly, we have identified a substantial number of mobile fullerenes, predominantly in local areas of the quasicrystalline monolayer covered by the 8/3 approximant. Thus, similar 8/3 approximant structures should exhibit similar behavior. An 8/3 approximant structure would even be advantageous because of the periodicity of the 3^6^ vertex configurations and the mobile fullerenes.

## 5. Rotational switching   

**Figure 5:**
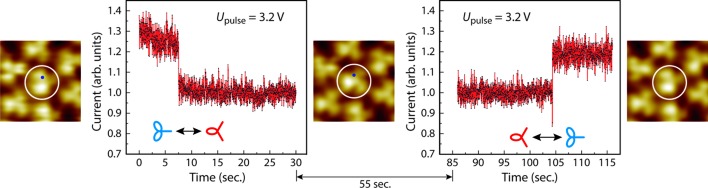
The change in the tunneling current above the central fullerene (circled in white) of a 3^6^ local structure of the QC monolayer. The blue dots in the STM images indicate the position of the tip. During the measurements, the feedback loop was turned off to maintain a constant tip-to-sample distance, while a bias voltage of 3.2 V was applied. The tunneling current changes are a result of the flip between two degenerate orientations of the central fullerene, as can be seen in the STM images (2.5 nm × 2.5 nm, *U*_bias_ = 2.22 V, *I*_set_ = 0.44 nA, slightly low pass filtered).

The ultimate proof that fullerenes at the center of a hexagon exhibit increased mobility compared with fullerenes in the 3^2^.4.3.4 vertex configuration is their flipping ability. Fig. 5 ▸ shows the flipping of a fullerene in the 3^6^ vertex configuration between two orientations, with an angle of 60° relative to each other. This rotational switching is induced by positioning the tip above the white-circled C_60_ in the left-hand STM image and applying a voltage pulse above a threshold value that induces rotation, *i.e.* 3.2 V for 30 s. A rapid change in tunneling current, in this case monitored after ∼7.5 s, indicates that a rotation of the C_60_ could be identified by scanning the same area again at a smaller sample bias voltage. The STM image in the center of Fig. 5 ▸ clearly shows that the white-circled C_60_ is rotated by 60° while the surrounding molecules have not moved. One reason for the drop in current is the position of the tip with respect to the orientation of the C_60_ cage. While the molecular orbitals rotate, the tip stays constant at the same position. At a positive bias voltage, higher tunneling currents should be measured if the tip is positioned above a pentagon, while lower tunneling currents should result if the tip is located above a hexagon. Due to the rotational switching of the central C_60_ by 60°, the position of the STM tip changes from above a pentagon to above a hexagon. By applying a second voltage pulse it could be shown that the rotational switching can be induced again. The tunneling current increases to its original value at the moment the molecule rotates. In the right-hand STM image of Fig. 5 ▸ the white-circled C_60_ again has the same orientation as in the left-hand STM image.

**Figure 6:**
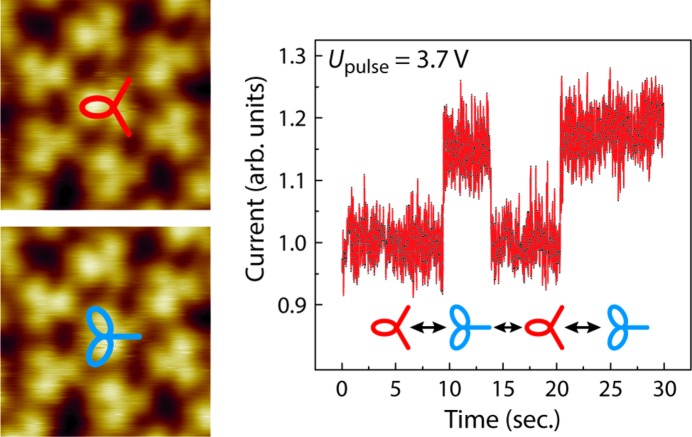
The current–time spectrum (*U*_pulse_ = 3.7 V) taken above the central C_60_ of a hexagonal local structure. (Left) STM images before (top) and after (bottom) applying the voltage pulse (3.0 nm × 3.0 nm, *U*_bias_ = 2.22 V, *I*_set_ = 0.59 nA).

**Figure 7:**
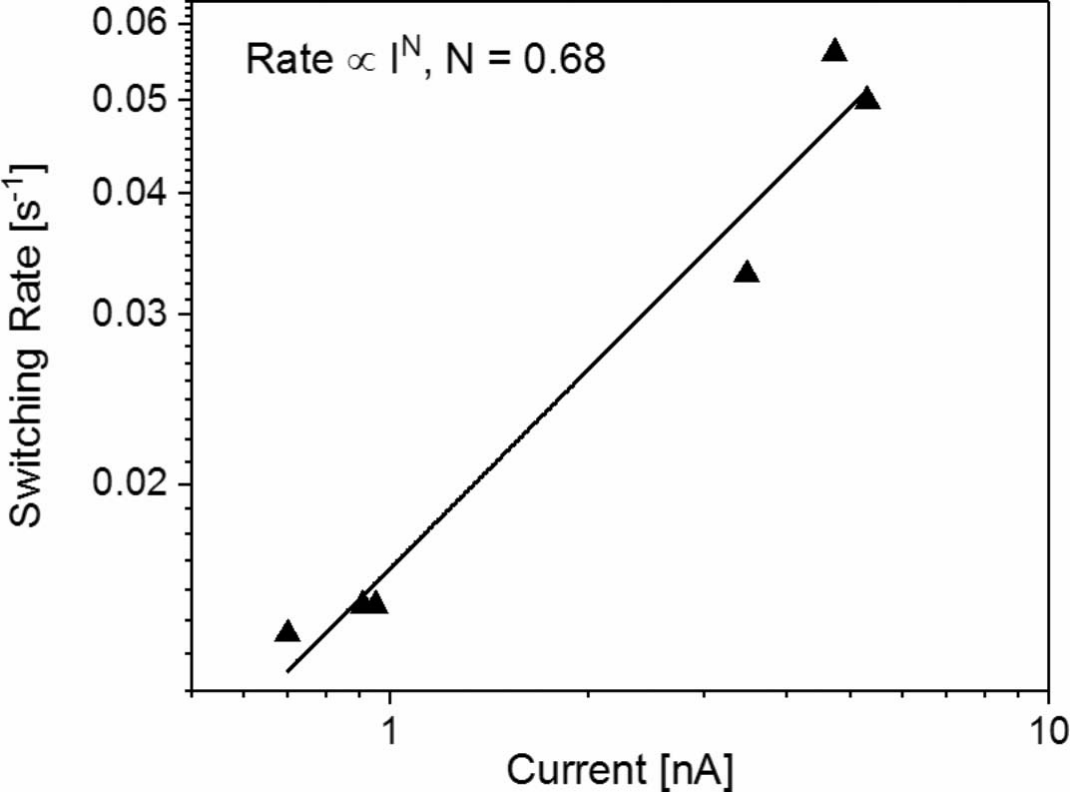
The rotational switching rate as a function of the tunneling current for fullerenes in the 3^6^ vertex configuration.

If an increased voltage pulse of 3.7 V is applied for 30 s to the C_60_ in the 3^6^ vertex configuration, multiple rotational switching events can be induced. In Fig. 6 ▸ three switching events can be identified in the current–time spectra. We performed more than 100 switching experiments at different tip heights and varying voltage pulses. Thus, the onset of the rotational switching of C_60_ in the 3^6^ vertex configuration was found at a threshold voltage of *U*_thres_ = 2.45 ± 0.2 V, corresponding to the energy alignment of the higher lying LUMO+3/LUMO+4/LUMO+5 states given by the conductivity spectra in Fig. 4 ▸. This voltage dependence shows that the rotational switching is resonantly enhanced by tunneling of electrons into empty states. Above *U*_thres_ we obtained an almost constant switching yield as a function of *U*_pulse_ up to 4 V. We avoided using higher voltages in order to prevent damage to the molecular arrangement. However, there was a slight decrease in the yield with increasing current, *i.e.* 2.8 ± 0.3 × 10^−12^ switches per electron for 0.85 nA and 1.6 ± 0.2 × 10^−12^ switches per electron for 4.5 nA. A higher current corresponds at constant *U*_bias_ to a smaller tip-to-sample distance. Thus, it can be suggested that the probability of inelastic scattering is reduced with the tip approaching the fullerene. Since the height of the tip over the fullerene is here in the distance regime of 0.5 to 1.0 nm, the reduced cross section of the scattering process through the fullerene might be the reason for the slightly decreased switching yield. In addition, we plotted the switching rate (*R*) as a function of the tunneling current in Fig. 7 ▸. From this plot *R* ≃ *I* ^*N*^, *N* = 0.68 was determined, which corresponds to the molecular switching behavior reported by Reecht *et al.* (2017 ▸). A power *N* = 1 would suggest a one-electron process. However, in our case we assume that the reduction in the switching yield is the reason for *N* < 1, while the rotational switching is a single-electron event.

It is well known that energy can be transferred from a tunneling electron to a molecule adsorbed on a surface and induce, for example, vibrational and/or rotational excitations (Reecht *et al.*, 2017 ▸). Since rotation implies some type of partial transfer of angular momentum, rotational excitation is more complex than vibrational excitation. Rotational excitation has been reported for copper phthalocyanine (CuPc) on Cu(111) (Schaffert *et al.*, 2013 ▸). However, the CuPc molecules undergo a frustrated rotational motion around their equilibrium position by flipping from an equilibrium position into a non-equilibrium position. In contrast, fullerenes in the 3^6^ vertex configuration perform a rotational switch between energetically nearly equivalent positions. From the crystallographic point of view, both of these rotational orientations are degenerate in adsorption energy. A slight difference in energy between the two orientations might be induced by changing Coulomb interactions with neighboring molecules (Lu *et al.*, 1992 ▸; Yuan *et al.*, 2003 ▸; Leaf *et al.*, 2016 ▸). However, the change in this stabilization energy due to rotation is expected to be insignificant, especially since the distance between neighboring fullerenes in the quasicrystalline structure is 1.04 nm. Furthermore, the three-lobe structures of the neighboring C_60_ surrounding the C_60_ in the 3^6^ local vertex configuration reveal that only a fraction of these C_60_ molecules have the same rotational orientation as the central C_60_, while the others are rotated by 60°. Consequently, the central C_60_ is, in each of the two possible rotational orientations, not able to optimize all of the nearest-neighbor Coulomb interactions. When the distribution of the rotational orientations of neighboring C_60_ is 3:3, like in Fig. 5 ▸, again both orientations of the central C_60_ are degenerate. Despite this fact, the central C_60_ with a rotational distribution of the neighbors of 2:4, shown in Fig. 6 ▸, and even in some cases 1:5, which is only rarely observed, can still be switched in most cases.

Rotational motion can be induced by the tunneling of electrons when the transferred energy is high enough to overcome the rotational barrier. If the STM tip is positioned above the fullerene in the hexagonal local vertex configuration and a voltage pulse (3.2 V, 30 s) is applied, an orientational switching can be induced in many cases. It should be noted that an application of the same voltage pulse, or even voltage pulses of up to 4.0 V, to C_60_ in the 3^2^.4.3.4 vertex configuration causes a rotational switching only on rare occasions. Thus, the switching ability of fullerenes on 3^6^ vertex sites is an intrinsic property of this dodecagonal monolayer and results in switching molecules well separated by a matrix of inert molecules.

## 6. Discussion   

**Figure 8:**
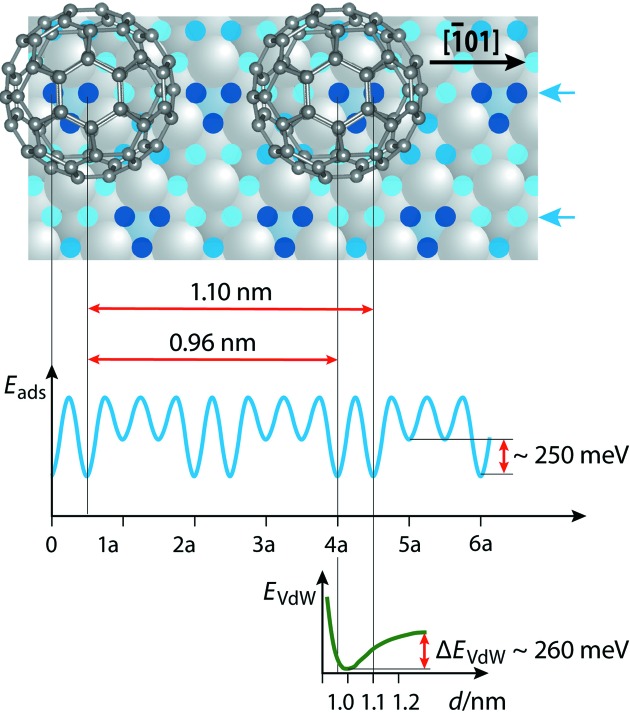
(Top) A schematic diagram showing the possible assembly of two neighboring fullerenes on bridge sites along the 〈
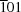
〉 directions on a 2Pt–Pt_3_Ti(111) surface, (middle) the adsorption energies (*E*_ads_) involved and (bottom) the van der Waals interaction energy (*E*_vdW_) (Gruznev *et al.*, 2013 ▸). The Pt—Pt distance is indicated by the letter a.

In order to gain further insight into the remarkable adsorption behavior of fullerenes on the 2Pt–Pt_3_Ti(111) surface, namely the formation of a quasicrystalline monolayer and the rotational switching of fullerenes on 3^6^ local vertex sites, we have to consider the interfacial interactions in detail. The adsorption-energy differences in the 〈
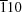
〉 directions, one of the nearest-neighbor directions in the dodecagonal monolayer, amount to 250 meV for different bridge sites (Paßens *et al.*, 2017 ▸). In Fig. 8 ▸ only one of the possible rows in this direction is given. This is an interesting row for the possible adsorption on preferred bridge positions, since two distances between neighboring fullerenes, 0.96 and 1.10 nm, can be realized. However, neither distance is optimal with respect to van der Waals interactions between fullerenes (Fig. 8 ▸, lower part) (Gruznev *et al.*, 2013 ▸). With an equilibrium intermolecular distance of 1.00 nm, a change to 1.10 nm would cost about 120 meV energy. Consequently, the fullerenes are tilted at a low energy cost in order to adopt the more preferred intermolecular distance of 1.04 nm as observed experimentally.

**Figure 9:**
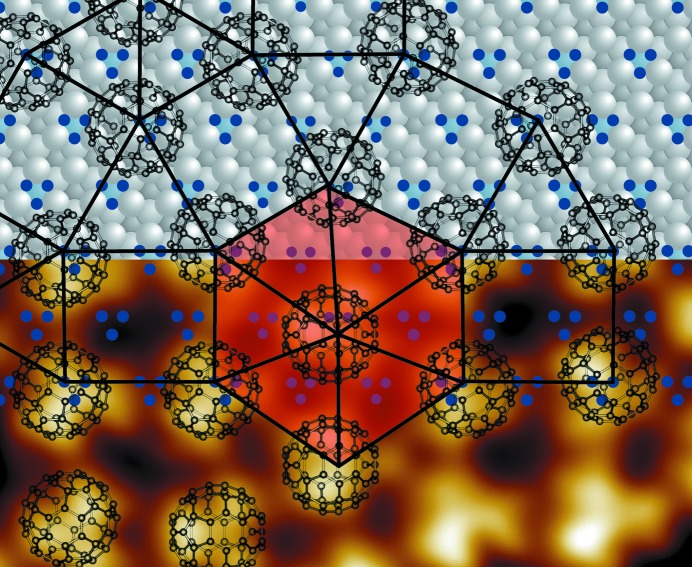
A schematic diagram showing the assignment of fullerenes to the energetically preferred bridge sites (dark-blue circles) on the 2Pt–Pt_3_Ti(111) surface. A hexagon in the superimposed tiling representation is highlighted. For details, see the text.

In the 〈
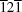
〉 crystal directions there is only one distance between preferred adsorption sites, namely 0.96 nm. This is rather short and obviously not realized in the quasicrystalline monolayer. However, if the direction is varied slightly (by 4°) other adsorption sites become available too, *i.e.* the three degenerate bridge sites within an area of 0.13^2^ nm^2^ in one surface unit cell (three dark-blue circles in Fig. 9 ▸, top part) allow for the adaptation of the intermolecular distance to some extent. Thus, only the energetically preferred adsorption sites are used by the fullerene molecules and the intermolecular van der Waals interactions are optimized by tilting. This can only be possible by using, in turn, the three crystallographically equivalent bridge sites within the area of 0.13^2^ nm^2^, but this constraint restrains the formation of a highly ordered monolayer with translational periodicity.

The second question is: why are the fullerenes in the 3^6^ vertex configuration more mobile than those on the 3^2^.4.3.4 vertex sites? To answer this question, we have analyzed the tilting of fullerenes belonging to a hexagon with respect to their adsorption positions (as shown in Fig. 2 ▸). The fullerenes are distributed on preferred adsorption sites in such a way that we utilize the available information from the STM image and add the other C_60_ arbitrarily. The assignment of the C_60_ to the adsorption positions is done with the constraint that the equivalent intermolecular distance of 1.00 nm should be met as closely as possible (Fig. 9 ▸). However, we did not try to figure out how much the real position of the fullerenes deviates from the preferred bridge position due to tilting. This information is not available from the STM images, since it is the fullerene surfaces that are mapped, not the interfacial structure, and this deviation should be of the order of 0.06 nm with an intermolecular distance of 1.00 nm. Furthermore, to determine the realized adsorption position exactly by theoretical means is a very complex problem involving many energy scales and multiple intermolecular interactions. Consequently, in the schematic shown in Fig. 9 ▸ (upper part), the fullerenes are related to adsorption positions without adaptation of their intermolecular distances to the experimentally observed 1.04 nm. Therefore, different intermolecular distances result in Fig. 9 ▸, in contrast with the experiment. Even though this schematic has its limitations, it is quite instructive. There are several possibilities for distributing the fullerenes among the different bridge sites but it is not possible to form a regular hexagon without further adaptation mechanisms like tilting. However, the central fullerene only has the possibility of optimizing the intermolecular distances to at most four of its six neighbors because of the symmetry constraints of the metallic surface determining the tilting direction. This situation is comparable with the geometric frustration problem in buckled colloidal monolayers discussed by Han *et al.* (2008 ▸). Thus, not all local interaction energies can be optimized simultaneously and the central fullerene will be frustrated. That means it reaches only one of several local minima but is thermodynamically not stable. On the other hand, a fullerene on the 3^2^.4.3.4 local vertex site has the ability to optimize at most four out of five nearest-neighbor interactions. Moreover, it is possible to achieve a symmetric environment of neighbors for the 3^2^.4.3.4 local vertex site on this 2Pt–Pt_3_Ti(111) surface. These arguments give some hints as to why the fullerene in the hexagonal vertex configuration is more mobile than others.

In conclusion, we have shown that the local potentials for the 3^6^ and 3^2^.4.3.4 vertex sites in the two-dimensional dodeca­gonal fullerene QC differ considerably. The fullerenes adsorbed in the 3^6^ vertex configuration exhibit a higher mobility than others, as verified by STM imaging, differential conductance spectra and the demonstration of voltage-dependent rotational switching. The geometric constraints imposed by the adsorption symmetry of fullerenes on the 2Pt–Pt_3_Ti(111) surface, as well as the interfacial and intermolecular energies involved, are possible reasons for the experimentally observed adsorption behavior. Moreover, it is deduced that the optimization of intermolecular distance-dependent van der Waals interactions between neighboring fullerenes degrades the realization of translational periodicity in the monolayer. It should be noted that the rotational switching fullerenes in this dodecagonal monolayer are separated from each other by inert fullerenes, and thus can be addressed individually by electrical pulses.
